# Population trends in the vulnerable Grey-headed flying-fox, *Pteropus poliocephalus*; results from a long-term, range-wide study

**DOI:** 10.1371/journal.pone.0298530

**Published:** 2024-03-21

**Authors:** Eric Peter Vanderduys, Peter Caley, Adam McKeown, John M. Martin, Chris Pavey, David Westcott

**Affiliations:** 1 Environment, Commonwealth Scientific and Industrial Research Organisation (CSIRO), Brisbane, Queensland, Australia; 2 Data61, Commonwealth Scientific and Industrial Research Organisation (CSIRO), Canberra, Australian Capital Territory, Australia; 3 Environment, Commonwealth Scientific and Industrial Research Organisation (CSIRO), Adelaide, South Australia, Australia; 4 Wildlife Services, Ecosure, Brisbane, Queensland, Australia; 5 Environment, Commonwealth Scientific and Industrial Research Organisation (CSIRO), Atherton, Queensland, Australia; University of Oklahama Norman Campus: The University of Oklahoma, UNITED STATES

## Abstract

Monitoring is necessary for the management of any threatened species if its predicament and status are to improve. Monitoring establishes baseline data for tracking trends in distribution and abundance and is a key tool for informing threatened species management. Across much of the Old World, bats in the genus *Pteropus* (Pteropodidae, Chiroptera) face significant threats from habitat loss, conflict with humans, and hunting. Despite conflict with humans and their threatened status, few *Pteropus* are being monitored. Often, this is because of difficulties associated with their high mobility, large and easily disturbed aggregations, and their use of unknown or remote habitat. Here we describe 10 years of results from the National Flying-fox Monitoring Program (NFFMP) for the grey-headed flying-fox, (*Pteropus poliocephalus*) in Australia. Range-wide quarterly surveys were conducted over a three-day period since November 2012 using standardized methods appropriate to conditions encountered at each roost. For our analysis of the population and its trend, we used a state-space model to account for the ecology of the grey-headed flying-fox and the errors associated with the surveying process. Despite the general perception that the species is in decline, our raw data and the modelled population trend suggest the grey-headed flying-fox population has remained stable during the NFFMP period, with the range also stable. These results indicate that the species’ extreme mobility and broad diet bestow it with a high level of resilience to various disturbance events. Long-term, range-wide studies such as this one, are crucial for understanding relatively long-lived and highly nomadic species such as the grey-headed flying-fox. The outcomes of this study highlight the need for such systematic population monitoring of all threatened *Pteropus* species.

## Introduction

Monitoring is a fundamental component of the management of wildlife [1]. It provides baseline data on key parameters such as species abundance, population trends, distribution, and habitat use. In the case of threatened species, this information is central to implementing conservation actions [2, 3]. Monitoring is particularly difficult for highly vagile species, and even more so when their movement occurs at scales of hundreds or thousands of kilometres [4–6]. Such movements vastly complicate monitoring efforts as they exceed the capabilities of local monitoring efforts and often cross jurisdictional boundaries, adding additional layers of complexity [7, 8]. Monitoring in such circumstances requires specific methodological designs that can accommodate the spatial scales and complexities involved in a large-scale study [9], and which are designed to deal with the additional errors introduced by the focal species’ ecology.

Flying-foxes, *Pteropus* spp. (Chiroptera, Pteropodidae), are an example of highly vagile animals with urgent monitoring needs [10]. The *c*. 60 flying-fox species are distributed from the western Indian Ocean, through India, southern Asia, Australasia, and into the central Pacific Islands. Most species (*c*. 47) are highly localised island endemics and restricted to one or a few islands [11–15] or have extensive island distributions or distribution on large islands (*c*. 11). Continental distributions are relatively rare (*c*. 8, one of which is extinct). Globally, flying-foxes are highly threatened; 31 extant species are listed as ‘vulnerable’ to ‘critically endangered’, with 22 of these having a decreasing population trend [16]. At least six historically extant species are now extinct [12, 16]. Threats include hunting pressure [e.g., 17, 18] loss of habitat, invasion by deleterious plants and animals, climate change [19], and human-wildlife conflict [summarised in 20, 21]. These threats often exacerbate low inherent fecundity [22] and population size [18, 23].

In spite of pressing conservation needs, there are few flying-fox monitoring programs worldwide. Monitoring of three Pacific Island species (*Pteropus samoensis*, *P*. *tonganus*, *P*. *mariannus* [24, 25]) has recently ceased. Elsewhere, several studies have informed population or subpopulation estimates, also of island species (e.g. *P*. *ornatus* and *P*. *tonganus* [26]; *P*. *rufus* [27]; *P*. *niger* [28] and *P*. *natalis* [29]). Critically, consistent long-term monitoring is required to establish meaningful population trends [30].

In Australia, flying-foxes usually roost communally during the day in aggregations numbering tens to thousands. Roosts occur in complex canopy vegetation and comprise groups of animals that are generally easily disturbed, and, consequently, are often difficult to count [30–32]. Furthermore, roost membership is not static; roosts represent a node in a network across the landscape [10]. Individual flying-foxes forage over tens, and sometimes hundreds, of kilometres a night and can change roosts frequently [32, 33]. This can result in dramatic changes in the size of individual roosts and the distribution of a species across its range over periods of days [34].

There are four mainland species of flying-fox in Australia, with two (grey-headed flying-fox (GHFF), *P*. *poliocephalus* and spectacled flying-fox, *P*. *conspicillatus*) listed species. Both listings are at least partially based on perceived declines in the populations, hence the need for monitoring. However, given their extreme mobility, both as individuals and populations, it is challenging to conduct whole-of-population monitoring [9, 30]. Since surveys of Australian flying-fox populations were first attempted, the difficulties in obtaining accurate numbers, and the biases involved, have been documented [35]. These difficulties in counting animals in roosts require methods that avoid or account for enumeration errors such as double counts or missing significant proportions of the population. Methods must also be repeatable. The National Flying-Fox Monitoring Program (NFFMP) is a continental scale program that monitors Australia’s mainland flying-fox species, with the explicit goal of establishing population estimates and trends of the GHFF and spectacled flying-fox. We have reported on the monitoring results for spectacled flying-fox elsewhere [30], and the background and implementation of the NFFMP are given in Westcott et al. [9].

While the latitudinal range of the GHFF appears to have changed little since the 1800s [36], the southern part of the range has tended to be occupied by summer migrants. Since the 1980s there has been a southerly expansion in roosts occupied year-round from Mallacoota to Melbourne, Victoria [37]. Since 2011, new permanent roosts established as a result of westerly expansion beyond Melbourne in Victoria, and as far west as Adelaide [38] and Port Augusta, South Australia. One of the main drivers for this is thought to be the diversity of winter foraging resources in urban areas [37, 39].

In this paper, we report on the population trend of GHFF. We use a state-space modelling (SSM) approach applied to quarterly monitoring conducted over a ten-year period across the species’ entire range. We do this to inform questions about population size and trajectory over the period of the NFFMP and compare this to pre-NFFMP survey data.

## Methods

### Study species

**Fig 1:**
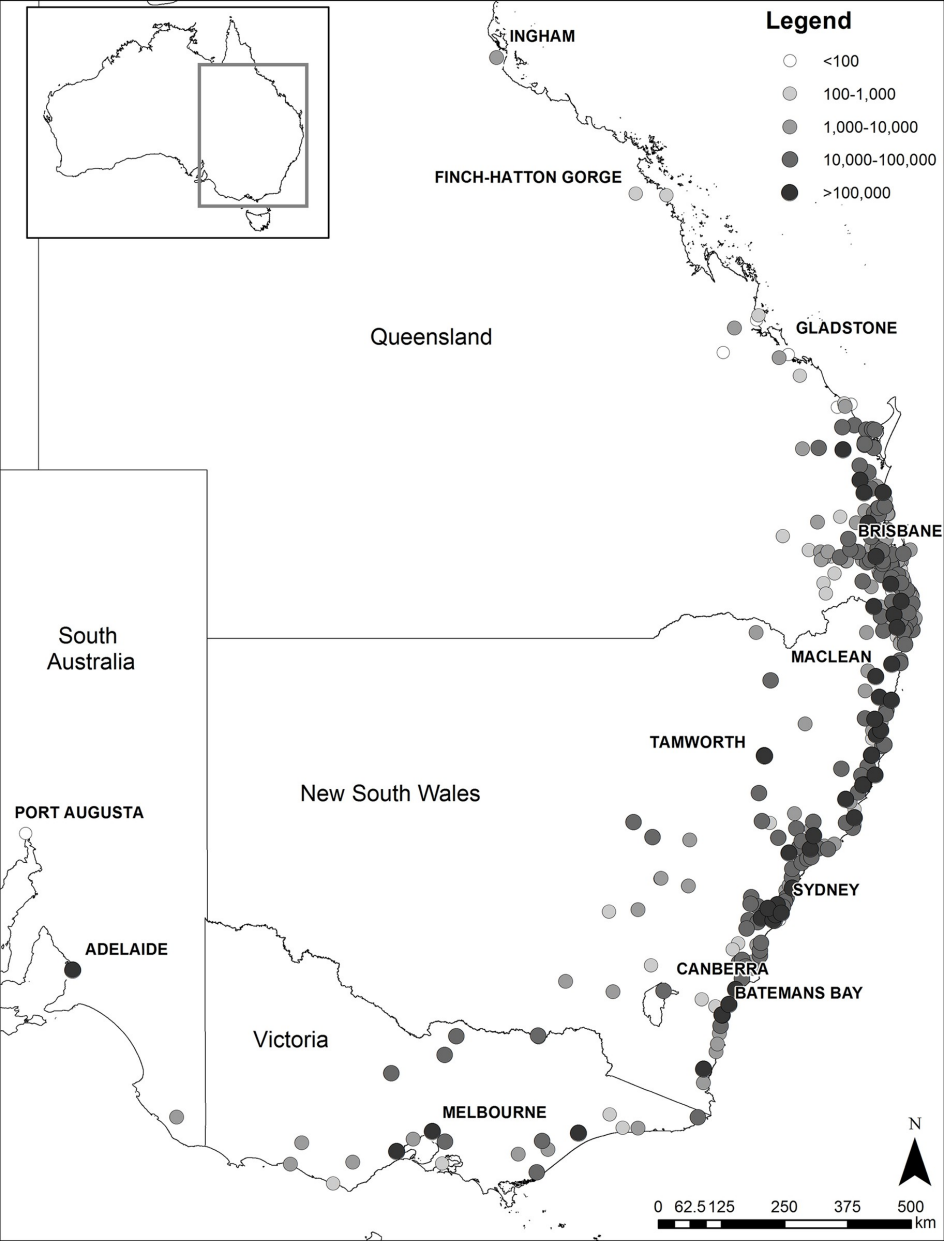
Map showing locations of camps surveyed with ≥1 record of GHFF in the NFFMP. Circle shade is indicative of cumulative numbers of GHFF per camp throughout the 10-year period of the NFFMP; darker circles = more GHFF recorded. Selected locations discussed in text, and capital cities are shown. Australian coastline and state boundaries used under Creative Commons Attribution 4.0 International license, sourced from https://data.gov.au/data/dataset/bdcf5b09-89bc-47ec-9281-6b8e9ee147aa/resource/71a50b42-ddca-422a-b1c7-50beb3306126/download/nov2023_adminbounds_gda_2020_shp.zip. Figure used CC BY 4.0 license, with permission from lead author Eric Vanderduys, original copyright 2024.

The GHFF is a large (body mass 410–1270 g; forearm length 164–177 mm [40]) Pteropodid bat, distributed from Port Augusta, South Australia to *c*. Gladstone, central Queensland, with an outlying roost in Ingham, north Queensland, and scattered roosts in between [34; this study; Fig 1]. The GHFF is listed as Vulnerable under Commonwealth legislation, and in the IUCN Red List [16, 41]. These listings are based on declining numbers in the order of 30% from 1989 to the period of 1998 to 2001, shrinkage of distribution, loss of habitat and probable competition and hybridisation with *Pteropus alecto* [41, 42].

GHFF are highly mobile, with individuals potentially travelling > 1000 km seasonally and up to 500 km in a 48-hour period [10, 43–45]. Nightly foraging forays by GHFF extend out to *c*. 148 km from their roost, though most movements are less than 30 km from the roost at their maximum distance [9, 38, 46].

GHFF are primarily florivorous, with Myrtaceae and Proteaceae being heavily targeted [47–49]. A variety of fruits, including native figs and orchard fruits, are less frequently targeted [40, 49]. They mate seasonally, around March–April [50] with *c*. six months of gestation and young supported by 3–4 months of lactation. Females generally give birth to a single pup per year [22, 51].

GHFF roosts may be used repeatedly over many years, often seasonally and less frequently, year-round [43, 44, 52, 53].

### Monitoring program

A population monitoring program of sufficient spatial and temporal resolution was both an objective and an action recommendation of the *Draft National Recovery Plan for the Grey-headed Flying-fox* [54]. The subsequent rationale, methodology and assumptions underpinning the NFFMP are detailed in previous reports [9, 32, 55] and are described here only briefly as they are applied to the GHFF.

GHFF were surveyed on a quarterly basis, every February, May, August and November from November 2012 to August 2022. Thirty-nine GHFF surveys were conducted in this period, with one cancelled (May 2020) and four constrained in extent by Covid-19 travel restrictions (August 2020, and February, May and August 2021). During the survey period GHFF were recorded in 507 distinct roosts, while in each quarterly survey they were recorded in a mean of 110 roosts (min 46, max 157). In addition, the NFFMP counts were compared to previous GHFF counts conducted in 1989 [56] and from 1998–2005 [57–60]. We make this comparison with caution because of both different methods employed and recommendations not to use those data for population estimates [41, 57, 58]. Despite the recommendation for caution, the previous counts need some consideration because it was an inferred population decline of *c*. 30% from those counts that made GHFF eligible for listing as Vulnerable [41], and previous population estimates have been inferred from those data [60, 61]. We also compare pre-NFFMP roost occupancy with the NFFMP roost occupancy to assess long-term (>20 years) roost occupancy. Pre-NFFMP counts were not used in any of the population modelling presented in this study; only the raw NFFMP data were examined.

For each survey, staff from CSIRO (Commonwealth Scientific and Industrial Research Organisation), relevant state government agencies, local government, community groups and individual volunteers were identified and assigned to count one or more roosts. Wherever possible counters were trained in each of the counting methods (see below) in face-to-face training sessions and provided with a training manual. Where training was not possible, due to location or limited time, counters were provided with the training manual. Effort has been invested in retaining counters in the program through feedback in the form of reports and data visualisation [see 62]. Training occurred in the weeks leading up to a count and most counters received training at least once during the program.

### Roost prioritisation method

Because the number of known GHFF roosts exceeded the availability of counters in any given quarterly survey period, roosts were assigned a priority prior to each survey. The intention was that if a roost had to be omitted because of a lack of counters during the survey, then it should be a roost that was expected, or known, to be small or unoccupied (typically class 0 or 1, see below). Effort was made to count flying-foxes in all the higher priority roosts in every survey. This prioritisation enabled the surveys to cover the greatest possible proportion of the GHFF population. Priority categories were assigned from 0 to 3 with Class 3 being the highest priority for counting and class 0 the lowest. Prioritisation was as follows:

- Class 3: roosts that had ≥10,000 GHFF in the previous two years, or ≥2,500 individuals present in at least four of the preceding eight quarterly surveys;
- Class 2: roosts that had any GHFF in the preceding two years;
- Class 1: roosts that had GHFF recorded during the last ten years, but not in the preceding 2 years;
- Class 0: roosts where GHFF had been observed historically, but never during any of the survey periods.

Because it could take up to two years for a roost to be classified as 1 or 2, roost classes were not necessarily assigned prior to November 2014 (i.e. eight surveys into the NFFMP). The prioritisation process described here was designed to enable the best deployment of limited numbers of field staff during quarterly surveys. For this reason, it is somewhat different to the definitions of “nationally important" roosts, which use the same number thresholds, but over a longer time frame [10 years; 63]. In our analyses, we only consider the classes defined above.

In the weeks leading up to the survey, roosts were checked where possible, and local observer knowledge accessed to determine occupancy and size, to aid the prioritisation process. New roosts found by local contacts or by GHFF tracking projects were included in the roost list and then prioritised using the process described above.

Counts were restricted to a three-day period to minimise sampling bias through double counting or missing individuals. GHFF are highly mobile [10] which can result in overnight changes in the size of a roost and the distribution of the species.

### Counting methods

Individual roost surveys (“counts”) were conducted by visiting daytime roosts, in preference to at dusk when the bats fly out to feed. Daytime counts allows many more roosts to be assessed, because counts can be conducted any time from dawn to the late afternoon, whereas fly-out counts are restricted to a limited time window each day, *c*. 40 minutes at dusk meaning that a very large number of volunteers are required to adequately cover the roosts within GHFF’s range [32, 55, 60, 64]. Daytime counts also allow species identification more easily than fly-out counts.

Differences in the accessibility, vegetation structure and behaviour of the flying-foxes mean that no single count method is appropriate for all roosts or even for a single roost on every visit. Consequently, a counter needs to assess the conditions at a roost and choose an appropriate method from one of five options. These methods are outlined in detail in previous publications [32, 55], but a brief description is provided below.

1. Distance sampling [65]. This technique requires the observer to be able to move through the roost without disturbing the animals. Standard distance sampling is employed at ≥ 15 random points throughout the roost, using groupings of animals, to the nearest metre, to a maximum of 20 m. The area of the roost is then recorded to determine the population. Because counters found this technique to be complex and intimidating, we only used this technique under permissive circumstances: large, relatively accessible roosts where the flying-foxes were not easily disturbed, and the counter was an experienced observer.
2. Area count. This method was employed in situations where the bats were easily disturbed by the observer. It involved counting the number of bats in a defined area, e.g. bats/m^2^ and averaging a large number of these counts across the roost footprint.
3. Tree count. Similar to an area count, however the average number of animals in a selection of individual trees was calculated, and the average number of individuals in these trees was extrapolated across the trees in the roost to provide an estimate of the roost size.
4. Direct count. When numbers were low, generally in roosts of < 1,000 individuals, and the bats were relatively visible, that is, not obscured by thick vegetation or at the tops of very tall trees, a direct count was conducted.
5. Fly-out counts [64]. If the roost was inaccessible with no suitable vantage points from which to view the animals, a fly-out count was used. This involved organising a group of observers to count the animals flying-out from the roost at dusk. This was used as a last resort technique, as it requires significantly more personnel, only one roost can be counted each day and a ground count is still needed to be conducted to assess the proportion of each species present.

Counting methods and surveys were approved by the following Commonwealth Scientific and Industrial Research Organisation (CSIRO) Animal Ethics Committees (AEC) and legislating departments;

- CSIRO Ecosystem Science AEC: approval number 13–02,
- CSIRO Wildlife, Livestock and Laboratory Animal AEC: approval numbers 2016–08, 2019–15,
- CSIRO Wildlife and Large Animal AEC: approval number 2019–04,
- Queensland Department of Environment and Science permit173P,
- New South Wales Office of Environment and Heritage licence SL101050.

The majority of roost sites were located in publicly accessible spaces where permits were not required.

### Population modelling and error estimation

Our modelling of the GHFF population (below) accounted for potential errors in the quarterly counts which are detailed in Westcott et al. [32], and summarised here.

First, not all GHFFs will be in known roosts during each survey period, either because they are roosting alone or at an unsurveyed roost. To quantify this metric, we follow Westcott et al. [32] who used GPS tracking technology (Camazotz tags, see [66]) attached to 68 individual GHFFs captured at six roosts across the range of the species. They found the proportion of days that GHFFs spent in known roosts was 89%, on average, based on 52,835 fixes from the tracked bats. This varied seasonally: February 100%, May 83%, August 73% and November 98%. The population estimates derived from the raw counts were corrected for these percentages [31]. Second, all survey methods have an associated error. We used repeat counts and comparisons of counts at the same roost but using different methods to estimate the accuracy and precision of the different methods [32]. These analyses suggest that daytime roost counts are both more accurate, and more precise, than fly-out counts [32, 64]. Third, we attempted to minimise errors because of missing occupied roosts by our prioritisation method (above).

#### General

The state-space model includes key features developed in Westcott et al. [30] when modelling dynamics of spectacled flying-foxes. These include: seasonality in the proportion of the total population that is within roosts and available to be counted; seasonal recruitment of young into the countable population; uncertainty applying to the demographic processes of mortality and recruitment; observation uncertainty associated with counts in individual roosts; and a random effect to account for variation in the proportion of individuals within roosts; and informative priors on all parameters if possible. As the model explicitly accounts for the temporal dependence (hence serial auto-correlation) in observations, it provides robust inference around population trend. This differs from the more traditional analysis of simply regressing the log of population density on time and testing for whether the slope coefficient is less than zero. Humbert et al. [67] show that the strength of inference of such analyses can be erroneous. Population modelling was not applied to the pre-NFFMP data [57–60], because the different methods they applied do not fit the assumptions of our modelling presented here.

#### Model detail—Mechanics

The underlying (latent) model assumes exponential population growth. That is,

$$\log\;{(N}_{t})=\log\;(N_{t-1})+\;r_{t}$$

where the rate of growth (*r*_t_) in between survey periods is a combination of the rate of recruitment into the counted population (ρ) and the rate of mortality (μ). Recruitment into the counted population was assumed to occur during December–February. Mortality of the countable population was assumed to occur year-round. This was modelled as

$$r_{t}=I_{\lambda }(t)\lambda +\mu$$

where *I*_λ_(*t*) is the indicator function that takes on the value 1 during December–February, and 0 otherwise.

The seasonality in the proportion of the population that is present within roosts (p_*t*_) is modelled using a constant value near 1.0 during January–February combined with a cosine function between March and December:

$$p_{t}=\frac{cos(2\pi t^{*}/12)+a_{1}}{a_{1}+a_{2}+1}$$

where t* is calculated from the calendar month (*t*_*mth*_) as

$$t^{*}=\frac{11(t_{mth}-2)}{12},$$

and parameters *a*_1_ and *a*_2_ determine the mean minimum and mean maximum proportions of the population within roosts. The number of individuals available to be counted in roosts is:

$$n_{t}=p_{t}N_{t}$$

and from this, the size of the population (*N*_*t*_) derived. Time accounting is undertaken monthly.

#### Model detail—Statistics

The structure of the model is set up to make inference on the underlying trend in population size, after accounting for sources of variation. The state-space modelling approach facilitates the incorporation of observation error (in the GHFF counting methods) and process noise. The sum of the counts of GHFF in all roosts were modelled with log-normal observation error:

$$\begin{array}{c} \log(n_{t})={\log(p}_{t}N_{t})+V,\mathrm{where}\\ V∼N(0,\sigma_{V}^{2}). \end{array}$$

Furthermore, we included process noise around the rate of recruitment, mortality and the proportion of the population within surveyed roosts during each GHFF national survey. The parameters and their prior values are detailed in Table 1. The prior values chosen implicitly generate a triangular-shaped distribution with a mean exponential rate of increase of 0.00 yr^-1^ (95% C.I. -0.27–0.26 yr^-1^).

**Table 1:** Model parameters, their description and prior values (including rationale).

Parameter	Description	Prior value^a^	Rationale
Recruitment (ρ)	Monthly rate of recruitment into countable population over 3-month period.	*U* (0, 0.34/3)	Uniform up until the maximum biologically possible [see also 22]
Mortality (μ)	Monthly mortality rate of countable population.	*U* (0, 0.69/12)	Maximum decrease population halving each year (excluding recruitment)
Observation error	The observation error applying to summed counts of number of individuals in roosts.	Lognormal (0, σ_V_)	Coefficient of variation “flat” from 5 –*c*. 50%, spanning what would be considered reasonable [32].
		σ_V_ ~ *U* (0.05, 0.50)	
Proportion of population in counted roosts	A flexible smooth seasonal function describing the proportion of the total population in counted roosts.	a_1_ ~ *U* (2, 12)	Informed prior based on radio-tracking data of [32]. See text and Appendix for more details.
		a_2_ ~ *U* (0, 0.2)	
Process noise around combined recruitment and mortality	Variation in rate of increase arising from unmeasured factors.	Normal (0, σ_W_)	Largely uninformative, with the CV around monthly rates of increase as high as 10%.
		σ_W_ ~ *U* (0.0, 1/12)	
Process noise (proportion in roosts)	Additional variation around the proportion of the total population within roosts (a seasonal random effect).	Normal (0, σ_P_) on logit scale where σ_P_ ~ *U* (0,2)	Reasonably “flat” when back-transformed to a probability from the logit scale [68]

^a^Distribution shorthand: “*U*” = Uniform

Models were fitted using the JAGS software [69] implemented in the R software environment [70]. Chains were checked for convergence using the convergence statistic of Gelman & Rubin [71].

From the posterior distribution, values for ρ and μ can be drawn jointly and used to calculate the underlying exponential rate of increase (*r*) for the overall population. Similarly, the estimated size of the population at different time points can be compared.

#### Survey precision and power analysis

A key purpose of monitoring programs such as the NFFMP is to detect changes in the population, which leads to questions of statistical power. That is, given a monitoring program, what is the probability that an analysis of the data will detect a specified change in population size as “significant” if it is truly occurring. The change can be expressed in absolute terms, and can be time-bounded. To undertake a prospective power analysis for the type of model we have fitted here (including multiple observations per year) is beyond the scope of this paper. However, for comparison, we undertake a power analysis similar to that of Westcott et al. [31] who evaluated the ability of once-yearly monitoring to detect an underlying trend consistent with a 30% population reduction over 10 years as statistically significant (at the 0.05 level). This timescale and accompanying reduction qualify a taxon for listing as Vulnerable under criterion A of the IUCN Red List, an approach that is used under Australia’s *Environment Protection and Biodiversity Conservation Act 1999* [41, 72].

Like Westcott et al. [31], we undertake a log-linear regression of population size (sampled yearly) on time, and assess the significant of the slope coefficient. This approach, though widely used (e.g. Eberhardt and Simmons [73]), has limitations [67]. However, we added process noise in the simulations using our model-derived estimates for the process noise (lognormally distributed with median *σ*_W_ = 0.03 mth^−1^). The observation error effectively used in Westcott et al. [31] was a combination of “population error” (CV = 20%) compounding with either “fly-out” (CV = 21%) or “observer” (CV = 10%) errors depending on the roost size and location. We used our model-based estimate for the observation error of the summed counts to be lognormally distributed with *σ*_V_ = 0.18. We note that the high coefficients of variation (CV) reported by McCarthy et al. [74] for a selection of ground counts apply to a single count only and so are unsuitable for applying to survey wide variation in the total count. The CV for the sum of estimates is invariably lower (i.e. better) than the CV for the individual estimates, as generally speaking, the standard error of a sum of *n* counts scales with the square root of *n*, so the CV of the sum scales by a factor of $1/\sqrt{n}$. We assume that all counts are taken during February when the proportion of the total population within roosts is near 1.

When taking the approach of Westcott et al. [31], each simulated population takes exactly the same downwards trajectory consistent with a 30% reduction over 10 years (i.e. r = log(0.7)/10 = −0.036 *yr*^−1^), and only the observations vary according to random draws consistent with the observation error. When process noise is included in simulation runs, a range of outcomes can occur due to the stochastic nature of population change, including population increases–despite the underlying population growth rate being negative. Assessing the power of an analysis to detect a population reduction that hasn’t occurred is largely nonsensical. To avoid this, we calculate the power at each year to simulated monitoring, conditional on a population decline of 30% or more having occurred. The results (see S1 Fig) indicate approximately 12 years elapse before a decline of 30% or more can be detected with 80% certainty at a significance level of 0.05. We expect that the power of the enhanced monitoring program (4 c.f. 1 observation per year) will be a significant improvement on this approach, and we will report on this in a separate publication.

We note that the purpose of power analyses is largely prospective (and arguably “frequentist”). In our case, the Bayesian model can provide us with a measure of the belief that the population has declined by a specified amount.

## Results

### Survey results

Surveyors conducted 11,939 visits to 912 roosts during the 39 NFFMP surveys from November 2012 to August 2022. GHFF were present and counted during 4,095 of these visits at 469 occupied roosts. Four hundred and forty-three roosts visited were unoccupied by GHFF during any NFFMP survey.

**Fig 2:**
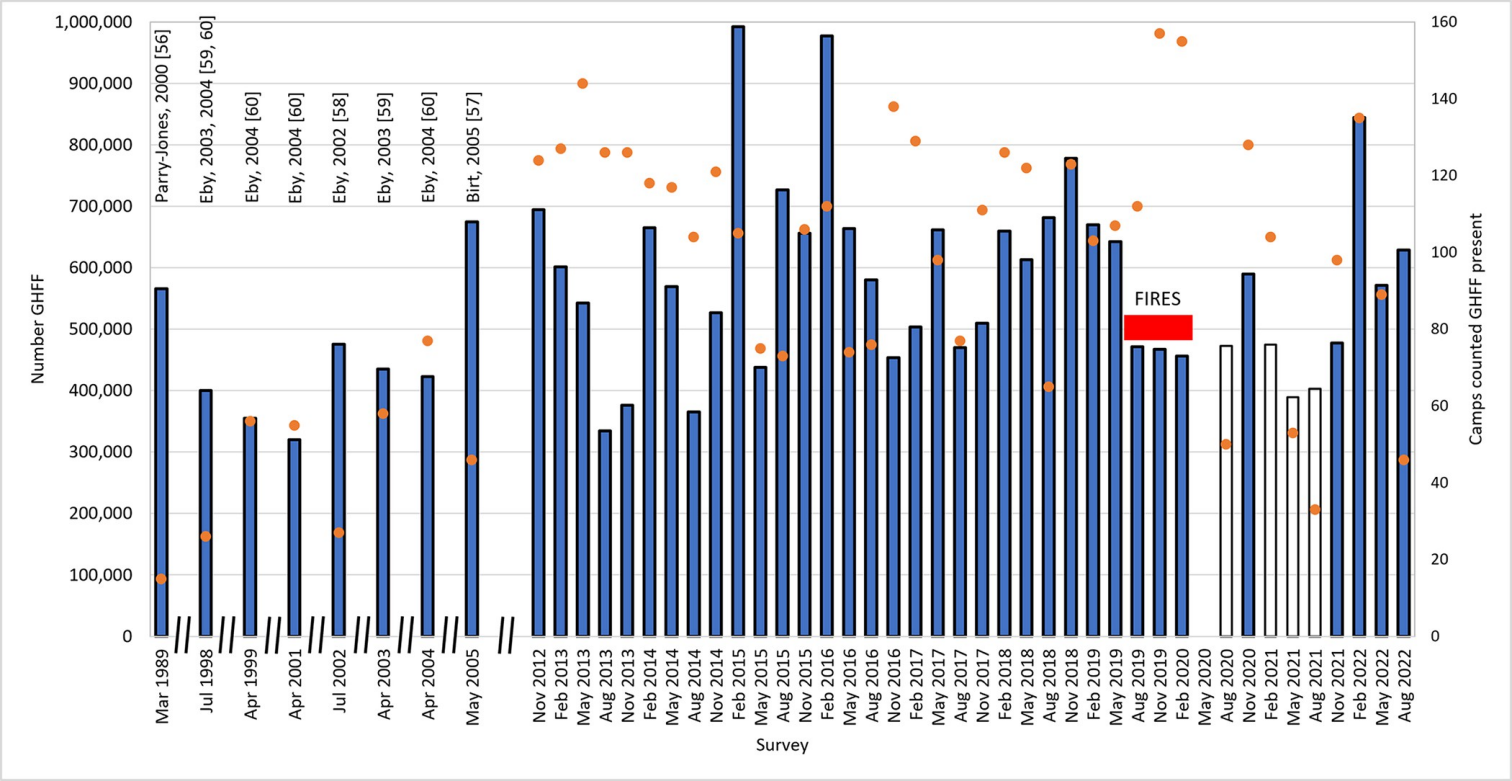
Raw counts of GHFF surveys from 1989 –August 2022 (blue bars). Counts prior to November 2012 are pre-NFFMP data (see text for sources). The open bars in August 2020 and February to August 2021 are absolute numbers from incomplete (Covid affected) counts. No counts were undertaken in May 2020. The duration of the megafires that burned from 1 July 2019 to 31 March 2020 [75] is indicated. These affected access to camps and resulted in thick smoke of many parts of the GHFF range. Note subequal X axis prior to the commencement of the NFFMP. Orange dots are the number of camps counted each quarter that had GHFF present.

The average raw GHFF number across all surveys was 578,350 (s.e. ± 24,445; range: 334,280 in August 2013, to 992,285 in February 2015). The largest single count was at Tamworth, NSW in August 2022, with *c*. 302,250 GHFF counted. Pre-NFFMP raw data from 1989 had a count of 566,000 individuals [56], and from 1998–2005 between *c*. 320,000–674,000 GHFF, from 15–229 roosts [57–60], to infer an “accepted [62] estimate … somewhere between 320,000 and 435,000 individuals” [61]. Raw survey numbers from the NFFMP and pre-NFFMP surveys are presented in Fig 2.

For high priority roosts, that is, those in classes 3 and 2, correct survey prioritisation cannot be guaranteed until November 2014. This delay is because of the requirement to collect baseline data in order to classify a roost into a class, as defined in the *Roost prioritisation method* section. The period November 2012 –August 2014 is not considered in calculating the change in numbers of class 3 and 2 roosts over the period of the NFFMP. The raw numbers of class 3 roosts fluctuated, but overall decreased fractionally, from November 2014 –August 2023 (R^2^ = 0.133), while class 2 roosts counted decreased slightly. Proportions of the less “valuable” class 1 and 0 roosts fluctuated more but overall, they decreased.

Not counting the first eight surveys because of the lag on calculating the class, there were on average 58 (min 46, max 72) class 3 roosts counted in each survey period. Of these an average of 85% (min 72%, max 96%) were surveyed each period.

### Long-term roost occupancy

Because of logistical limitations during the NFFMP there were only six roosts that were counted in every survey. Therefore, to assess probability of continuous occupancy we looked at roosts that had been surveyed in at least 30 of the 35 full surveys (84 roosts). Of these, there were six (7.1%) roosts that were continuously occupied by GHFF in every survey of the roost conducted; Parramatta Park, Lismore-Rotary Park, Maclean, Balgowlah, Centennial Park (NSW) and Adelaide (SA). However, there were 45 roosts (9.6%) that were occupied in at least one survey in every year of the program.

From the pre-NFFMP counts, we identified 104 roosts occupied by GHFF between 2003 and 2005 [57, 59, 60], and of those 74 (73%) were also occupied by GHFF during at least one NFFMP survey. Of the six roosts that were continuously occupied during the NFFMP, none were continuously occupied during the 2003–2005 surveys.

### Change in population

**Fig 3:**
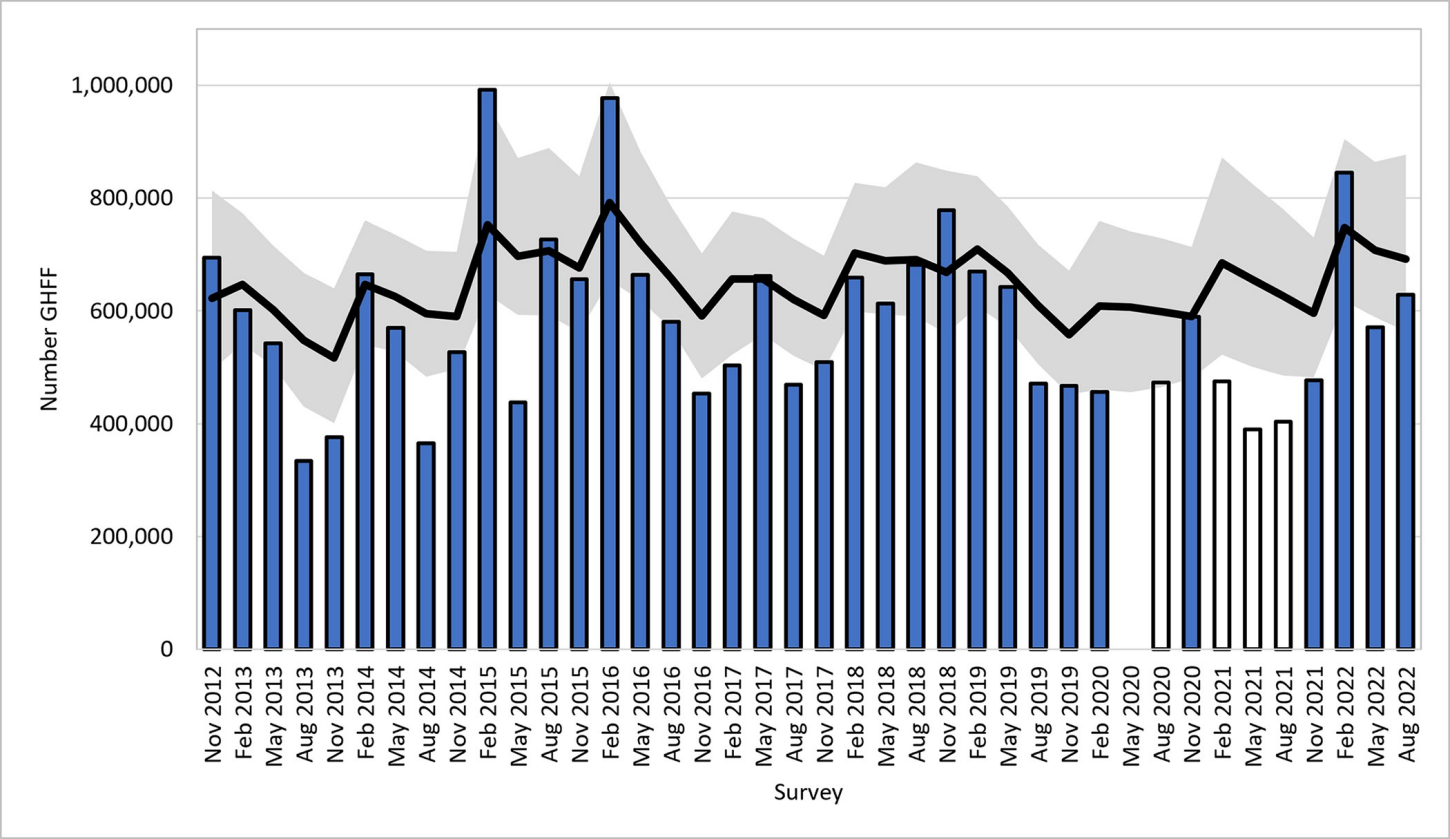
See previous figure for different bar symbology. NFFMP raw survey counts with modelled population included. The grey shaded area represents the 95% credibility interval around the median (black line) of the population estimate. The open bars were not used in the model. No counts were undertaken in May 2020.

When examining the posterior estimates for the total population size at each survey time, we find that there is a 72% probability that the final (August 2022) population size (median = 692,000, 95% C.I. 563,000–877,000) is greater than the initial one (November 2012) (median = 622,000, 95% C.I. 496,000–813,000; Fig 3). That is, there is an approximate 2.6 times greater confidence that the population has increased than decreased. Furthermore, we can also use the posterior distributions of population size to estimate the chances of the population having decreased by a specified amount. We find that there is a 0.9% chance that the August 2022 population has declined by at least 30% in comparison to the November 2012 population.

From the posterior distribution for recruitment and mortality, the estimated rate of increase of the underlying population model has a median of 0.003 yr^-1^ (95% C.I. -0.17–0.14 yr^-1^)–there is no evidence for increase or decrease (*r* = 0 is equivalent to a finite rate of λ = 1). From the posterior distribution of the rate of increase (see S3 Fig), there is a 24.7% chance that the underlying rate of increase is less than -0.036, which is the rate that would be consistent with a 30% decrease in the population over a 10-year period.

## Discussion

We found that the Vulnerable GHFF population was relatively stable over the 10-year survey period. The quarterly counts fluctuated considerably, but our consistent long-term data shows that these fluctuations did not necessarily represent population changes, but expected variation in survey effort, accuracy and precision. From the modelled data, accounting for survey error, the estimated underlying rate of increase (r = 0.003 yr^-1^, 95% C.I. -0.17–0.14 yr^-1^) suggests a stable population from *c*. 622,000–692,000. Our forward projection model predicts that adding three years of quarterly survey data would increase the monitoring power to 80%, and an additional six years would increase it to nearly 90% (S1 Fig).

Our result of a stable GHFF population over the past 10-years provides grounds to be optimistic for this species, especially given the context that there has been ongoing habitat clearing [76–78] and wildfires burning large amounts of feeding habitat [79–81]. There have also been severe heatwaves that have killed tens of thousands [82–85] of flying-foxes, including GHFF. Our finding of a stable population contradicts the findings of earlier, less comprehensive, annual surveys. For example, in 1989, a count of 566,000 GHFF was made at 15 roosts [56], and from 1998–2005 between *c*. 320,000–674,000 GHFF were counted at 26–77 roosts [57–60]. The variability reported between these counts would have been significantly improved, if not removed, if consistent long-term population-wide data had been collected.

Previous ‘national’ surveys of GHFF were not able to cover the whole spatial range of the species, nor its seasonal changes in roost populations due to time and management constraints. Species that are nomadic on a continental scale, such as GHFF [10] are likely to be particularly prone to perceptions of population change if their whole range is not accounted for. Our survey data also shows large seasonal changes in counted populations, and changes between years, so necessitating long term monitoring. In Australia, where myrtaceous plants can be episodic and unpredictable [86–88] in their flowering, any regional perceptions of change, must be countered with exploration of where the population may have moved to.

The magnitude of regional GHFF population change is useful to note to help understand difficulties in interpreting past survey data, and relating those data to the more consistently collected data of the NFFMP surveys. Birt [57] reported very large numbers (>220,000) of GHFF at Kioloa (“Kiola” [57]) in May 2005. This massive count and similarly large numbers in 1985 [89, 90] and 2016 (NFFMP data; this study) in the same region of NSW, are linked to mass flowering events of particular myrtaceous plants, especially spotted gum *Corymbia maculata*. Despite large numbers being present in one season or quarterly count, numbers can be completely depleted within a few months, and may not return the following year if the food resources are not available. For example, Parry-Jones [90] reported that there were no GHFF at in the area in 1986, and that *C*. *maculata* (as *Eucalyptus maculata*) had failed to blossom. Similarly, our NFFMP data suggested >270,000 GHFF in the Kioloa/Batemans Bay area in May 2016, and subsequent quarterly counts recorded from 0–400 GHFF for the following year, although this was exacerbated by management actions to disperse the roosts in the urban area. The single largest roost count, >302,000 GHFF in Tamworth in August 2022 contrasts with no GHFF observed at the roost a year earlier, and reportedly no GHFF in the roost one month after the August 2022 count (but after formal surveys were complete). These types of mass movements are typical and highlight the need for both regular and range wide monitoring if the population trend is to be understood.

Less than 2% of roosts were continuously used in the period of the NFFMP. No roosts were continuously occupied during both the earlier 2002–2005 surveys and the NFFMP period. This suggests that continuous occupation of individual roosts is uncommon over the medium term, and unknown over the long term. All the roosts that were continuously occupied during the NFFMP were in urban landscapes, potentially because of the attractiveness of these landscapes and their year-round food resources [38, 39, 46]. With the increase in urbanisation of the flying-foxes in Australia [33, 91], an increasing tendency for the species to form continuously occupied roosts in urban areas may develop. Almost 10% of used roosts were occupied during at least one survey in each year of the program, highlighting the nomadic nature of GHFF and the cultural or habitual use of certain roosts.

Individual roosts may not be continuously occupied, however roosts in the same vicinity probably serve the same functional role. For example, Maclean was continuously occupied during the NFFMP, but not during the 2002–2005 surveys. However, there were GHFF in all of these earlier surveys at the nearby Yaegl Nature Reserve roost, 2,500 m away. From a roost management perspective, they are different roosts with different tenures and amenity problems, and therefore need to be managed differently. However, from a GHFF ecological perspective, they most likely serve the same functional role—somewhere convenient to roost that allows access to the surrounding resources. From this perspective we can think of Maclean as being continuously occupied since 2002. Therefore, it is useful to think of close-proximity roosts as “constellations” of roosts when interpreting GHFF ecology.

These reasons—scale and temporal fluctuation—are what the NFFMP was designed to account for, and highlight the value of long term, range wide monitoring, especially for long-lived, nomadic species with large geographic ranges.

### Fires

The effects of recent (2019–2020) megafires on GHFF roosts were minimal. While there were accounts of some smaller roosts being affected, and direct mortality in one published case [92], the only major roost burnt out was Kioloa. However, this roost was being reused by GHFF by 2022. The minimal impact on the roosts is not surprising given the propensity of GHFF for roosting in low-lying, moist, humid gullies, and in proximity to humans. The fires burnt much potential GHFF feeding habitat in south-eastern Australia, but the 2022 survey results show no evidence for an effect on the population, despite the extent of the feeding habitat that burnt in 2019–2020 [81]. Roosts in the badly burnt areas on the south coast of NSW were still being used by large numbers of GHFF after the fires, indicating that there were still resources in these areas which they were able to access. While there is no evidence of any major food shortages for GHFF that may result from massive losses of food trees, it is unclear how long before the full recovery of the burnt food resources occurs. Myrtaceous plants within the range of the GHFF and that are food sources for the species exhibit varied flowering responses to fire [81, 87, 93], with some showing high levels of resistance and recovery [94]. These varied responses, combined with GHFF’s high mobility, breadth of diet, probably high adult survivorship year-to-year [56] and moderate life span [up to 18 years; average life span 7.1 ± 3.9 years; 95] should increase the chances of maintenance of a steady GHFF population post-fire.

### Heatwaves

In January 2019, there was a severe heatwave that caused considerable mortality in some GHFF roosts; *c*. 2000–3000 individual GHFF dead in Adelaide and *c*. 2300 in eastern Victoria [83, 84]. This was less than 1% of the estimated total population. Though pup deaths are specifically mentioned from Victoria [84], mortality data from this heatwave contains little demographic information but previous works suggest juveniles are the worst affected [22]. If juveniles are the most affected, then the outcome for GHFF populations will be less dire than if large numbers of adults die. Large numbers of adult flying-fox deaths have been reported as personal communications, but are unquantified [see 22, p. 80].

As temperature extremes increase with latitude [96], it is expected that there will be increasing occurrences of heat-stress events particularly in the southern range of the species [19]. As this is territory that has only recently been permanently occupied, these events may potentially cause a reversal of this expansion with the species retracting northwards.

We are concerned that ongoing heatwaves may affect GHFF populations dramatically in the future. This study is based on the first 10 years of the NFFMP. If heatwaves (and fires, discussed above) do start to affect GHFF population size, we expect this to take several years to begin to be detected by the methods we employ, which emphasises the usefulness of our methodology. Temperature records have recently been broken within the species’ range in SA, NSW and the ACT [97–99]. If this trend continues, mortality of adult GHFF may become a more commonplace occurrence.

### Isolated populations

There are two known isolated roosts of GHFF well to the north of the ‘typical’ range of the species (Fig 1). One is at Finch-Hatton Gorge, in central Queensland rainforest and the other is at Ingham in the Wet Tropics bioregion of north Queensland (as of July 2023, this roost was subject to dispersal activities and the whereabouts of the individual GHFFs from the roost are unknown). While the numbers of GHFF in both these roosts are generally low (Ingham: hundreds, NFFMP; Finch-Hatton: "hundreds to thousands", Parsons, 2010), the presence of these outlying roosts begs the question: why aren’t GHFF reported from other roosts between Rockhampton and the Wet Tropics? A hypothesis is that roosts in this intervening region may be in mangroves, and consequently difficult to survey. However, there are urban roosts of other flying-foxes in this region where we expect the presence of GHFF would be noticed. Only more carefully targeted monitoring of flying-fox roosts and tracking studies between central and north Queensland can address this question.

### Counter experience

Repeatability is critical for long term monitoring. Experience of the individual counters is a critical part of this repeatability. For this reason, it is imperative to retain observers, especially experienced ones, for as long as possible. Retention of experienced volunteers has long been recognised as important for monitoring programs such as for GHFF [9, 55, 57, 58].

### The future of GHFF

The trend for increasingly severe fire conditions is strongest within southern and eastern Australia [100, 101], overlapping almost entirely with the range of GHFF. Extensive reviews of fire or GHFF in Australia [102–106] and specific papers within [e.g. 107, 108] do not discuss survivorship of *Pteropus* spp. To our knowledge there is only one report of direct mortality from bushfire; that of an unknown number of probable GHFF at Jeremadra on the NSW south coast [92]. Our hope is that the methods employed in the NFFMP may help clarify GHFF response to wildfires of the scale seen in 2019–2020.

GHFF have been described as “sequential specialists” (Divljan (2008), p. 19), following changing and patchy food resources. Our results show there have been no declines in the face of what appears to be population insults (fire, drought, heatwaves). Perhaps it is precisely because of the unpredictable patterns of resources that GHFF evolved to exploit, that they appear to be such a resilient species. This is a species that readily moves across its range following food availability [10, 36, 44], often travels long distances each night to access these resources [32], and has a wide dietary range enabling it to work around losses of food resources from factors such as widespread wildfires. These characteristics indicate a species with a strong capacity for resilience, despite the loss of “critical winter habitats” [81].

The IUCN listing for GHFF is based on an inferred population decline to 467,000 GHFF in November 2019 [72]. This count was undertaken during the 2019–2020 megafires, which were heavily smoke- and access-affected. Subsequent counts recorded higher and lower numbers than this, but higher numbers (*c*. 590,000 in November 2020, *c*. 520,000 in November 2021 (a heavily Covid-restricted count, with just 33 roosts visited), *c*. 845,000 in February 2022) highlight the importance of intense monitoring, combined with informed population modelling, rather than picking a single count estimate as “the population”. We recommend the IUCN listing advice be reviewed using the information presented here.

Concern about a given bat species’ conservation status is common but conflicted [109]. There is often reticence about delisting threatened species. There may be concerns that the science underpinning such decisions may be flawed, or inertia and dogma regarding a species’ status or population trajectory might be at play. In this situation the status quo may be difficult to challenge. This can potentially result in resources being diverted to what might otherwise be lower priority issues, create conflict around management which might otherwise be avoided, and, in some circumstances, can obscure what might otherwise be conservation ‘good news’ stories. We want to avoid the situation presented by Garnett and Lindenmayer [110] where delivering bad news earns status points in the conservation community. Hopeful stories should be delivered when supported by evidence [111].

The data presented here are a good-news story. During the duration of the NFFMP, GHFF have almost certainly not declined. Rather, the population seems stable, or is possibly slowly increasing.

Prior to the NFFMP, surveys of the GHFF have only covered part of the range, or have had limited temporal resolution, which has led to uncertainty around overall population trends. Currently there are no range wide surveys being conducted of the GHFF, though some regions are reporting in results (to AM, project database manager). While this is useful for local roost management information, the current patchwork nature of the data highlights that without coordinated whole-of-range surveys it is impossible to gauge trends and overall movements of the population. Given predictions of increased extreme fire weather [112–114], and potentially heatwaves [115], which may act in concert, it will be necessary to continue monitoring the population in some capacity in order to determine what legislative response may be appropriate for this species.

Accurate population counts are critical for monitoring threatened species. They are important for informed management decisions about those species, including allocation of scarce management resources. The 2009 *Draft National Recovery Plan for the Grey-headed Flying-fox* [54] recommended that range wide regular surveys be conducted, and the creation and implementation of the NFFMP has realised this goal.

Technological advances since the inception of the NFFMP may mean that additional methods may be employed in any future monitoring program to increase precision and accuracy. For example, weather radar has been verified as a useful tool under certain geographic conditions and for certain flying-fox roosts [116]. These obviously cannot resolve species composition but may be a useful adjunct to visual count in the future. To the best of our knowledge, mobile, made-for-purpose bird and bat detection radars have not yet been used in Australia, but these offer the potential for further refinement of radar bat-monitoring [117, 118]. Thermal drone technology has also been employed allowing high degrees of accuracy and precision under certain circumstances, and there are significant skills, equipment, time and regulatory costs involved, and some ground-truthing is still necessary to discern species in most cases. These novel methods are likely to be increasingly effective and useful, particularly as image and video interpretation software (AI) improves.

## Conclusion

Our study demonstrates the value of a consistently collected, medium to long-term dataset for clarifying population trends in highly vagile species such as GHFF. It also shows that collecting quarterly data is a useful method for clarifying the “signal from the noise”. It suggests that the GHFF population has been more or less stable over the survey period of the NFFMP.

## Supporting information

S1 Fig Power to detect a minimum 30% reduction in population size occurring by year of monitoring. Results are derived from log-linear modelling of simulated total national counts of grey-headed flying foxes, where simulations assume an underlying decrease of 0.036% reduction / year, though the realised population growth is subject to process noise (see text for details).
(TIF)

S2 Fig Prior distribution for the seasonal variation in the proportion of the population that is within counted camps. The solid line represents the mean, and shading the 95% credibility interval. Observed datapoints represent the proportions of radio-tracked individuals within camp [32].
(TIF)

S3 Fig Posterior distributions for (A). Standard deviation of estimated log-normal count error, (B). Standard deviation of monthly process noise in population change, (C). Rate of recruitment into population during recruitment months (December, January, February), and (D) monthly mortality rate (year-round).
(TIF)

S4 Fig Posterior distribution for the exponential rate of increase for the population, overlaid on the implicit prior distribution. (TIF)

## References

[1] Nichols JD, Williams BK. Monitoring for conservation. Trends in ecology & evolution. 2006;21(12):668–73. Epub 2006/08/22. doi: 10.1016/j.tree.2006.08.007 . 16919361

[2] Robinson NM, Scheele BC, Legge S, Southwell DM, Carter O, Lintermans M, et al. How to ensure threatened species monitoring leads to threatened species conservation. 2018;19(3):222–9. 10.1111/emr.12335.

[3] Lindenmayer D, Woinarski J, Legge S, Southwell D, Lavery T, Robinson N, et al. A checklist of attributes for effective monitoring of threatened species and threatened ecosystems. Journal of Environmental Management. 2020;262:110312. doi: 10.1016/j.jenvman.2020.110312 32250795

[4] Perrig PL, Lambertucci SA, Donadio E, Padró J, Pauli JN. Monitoring vultures in the 21st century: The need for standardized protocols. 2019;56(4):796–801. 10.1111/1365-2664.13348.

[5] Olival KJ, Higuchi H, editors. Monitoring the long-distance movement of wildlife in Asia using satellite telemetry. Kathmandu, Nepal 2006.

[6] Mattsson BJ, Mateo-Tomás P, Aebischer A, Rösner S, Kunz F, Schöll EM, et al. Enhancing monitoring and transboundary collaboration for conserving migratory species under global change: The priority case of the red kite. Journal of Environmental Management. 2022;317:115345. doi: 10.1016/j.jenvman.2022.115345 35642814

[7] Legge S, Lindenmayer DB, Robinson NM, Scheele BC, Southwell DM, Wintle BA, et al., editors. Chapter 1. Introduction: making it count Clayton South, Victoria: CSIRO Publishing; 2018.

[8] Legge S, Scheele BC, Woinarski JCZ, Garnett ST, Keith DA, Lintermans M, et al., editors. Chapter 9. Summary: monitoring extent and adequacy for threatened biodiversity. Clayton South, Victoria: CSIRO Publishing; 2018.

[9] Westcott DA, McKeown A, Parry H, Parsons J, Jurdak R, Kusy B, et al. Implementation of the National Flying-Fox monitoring program. Rural Industries Research and Development Corporation Publication No 15/101 Project No 008241. 2015:1–190.

[10] Welbergen JA, Meade J, Field HE, Edson D, McMichael L, Shoo LP, et al. Extreme mobility of the world’s largest flying mammals creates key challenges for management and conservation. BMC Biology. 2020;18(101). doi: 10.1186/s12915-020-00829-w 32819385 PMC7440933

[11] Almeida FC, Giannini NP, Simmons NB, Helgen KM. Each flying fox on its own branch: A phylogenetic tree for Pteropus and related genera (Chiroptera: Pteropodidae). Molecular Phylogenetics and Evolution. 2014;77:83–95. doi: 10.1016/j.ympev.2014.03.009 24662680

[12] Helgen KM, Helgen LE, Wilson DE. Pacific Flying Foxes (Mammalia: Chiroptera): Two New Species of Pteropus from Samoa, Probably Extinct. American Museum Novitates. 2009;3646:1–37. doi: 10.1206/614.1

[13] Simmons NB. Order Chiroptera. In: Wilson DE, Reeder DM, editors. Mammal Species of the World: a taxonomic and geographic reference. 3 ed. Baltimore: The Johns Hopkins University Press; 2005. p. 312–529.

[14] Almeida FC, Simmons NB, Giannini NP. A Species-level Phylogeny of Old World Fruit Bats with a New Higher-level Classification of the Family Pteropodidae. American Museum Novitates. 2020;3950:1–24.

[15] Tsang SM, Wiantoro S, Veluz MJ, Sugita S, Nguyen Y-L, Simmons NB, et al. Dispersal out of Wallacea spurs diversification of Pteropus flying foxes, the world’s largest bats (Mammalia: Chiroptera). Journal of Biogeography. 2019;2019(00):1–11. doi: 10.1111/jbi.13750 33041434 PMC7546435

[16] IUCN. The IUCN Red List of Threatened Species. In: The IUCN Red List of Threatened Species. 2018–3. 2020.

[17] Graham GL, editor. Part 2. Population Threats. Conservation and Subsistence Harvesting of Pacific Island Flying Foxes. Washington: U.S. Fish and Wildlife Service; 1992.

[18] Wilson DE, Engbring J, editors. Part 3. Population Status. The Flying Foxes *Pteropus samoensis* and *Pteropus tonganus*: Status in Fiji and Samoa. Washington: U.S. Fish and Wildlife Service; 1992.

[19] Welbergen JA, Klose SM, Markus N, Eby P. Climate change and the effects of temperature extremes on Australian flying-foxes. Prceedings of the Royal Society B. 2007;275:419–25. doi: 10.1098/rspb.2007.1385 18048286 PMC2596826

[20] Vincenot CE, Florens FBV, Kingston T. Can we protect island flying foxes? Science. 2017;355(6332):1368–70. doi: 10.1126/science.aam7582 28360279

[21] Voigt CC, Kingston T, editors. Bats in the Anthropocene: Conservation of Bats in a Changing World. Switzerland: Springer Open; 2016.

[22] McIlwee AP, Martin L. On the intrinsic capacity for increase of Australian flying-foxes (Pteropus spp., Megachiroptera). Australian Zoologist. 2002;32(1):76–100. doi: 10.7882/az.2002.008

[23] Stinson DW, Glass PO, Taisacan EM, editors. Part 3. Population Status. Declines and Trade in Fruit Bats on Saipan, Tinian, Aguijan, and Rota. Washington: U.S. Fish and Wildlife Service; 1992.

[24] Brooke AP. Population status and behaviours of the Samoan flying fox (Pteropus samoensis) on Tutuila Island, American Samoa. Journal of Zoology. 2001;254:309–19.

[25] Utzurrum RCB, Wiles GJ, Brooke AP, Worthington DJ. Count Methods and Population Trends in Pacific Island Flying Foxes. In: O’Shea T, Bogan MA, editors. Monitoring trends in bat populations of the United States and territories: problems and prospects Information and technology report: USGS/BRD/ITR-2003-0003; 2003. p. 49–60.

[26] Oedin M, Brescia F, Boissenin M, Vidal E, Jean-Jérôme Cassan J-J, Jean-Claude Hurlin J-C, et al. Monitoring hunted species of cultural significance: Estimates of trends, population sizes and harvesting rates of flying-fox (Pteropus sp.) in New Caledonia. PLoS ONE. 2019;14(12):e0224466. doi: 10.1371/journal.pone.0224466 31891573 PMC6938311

[27] Hyde Roberts S, Jacobs MD, Clark RM, Daly CM, Tsimijaly LH, Rossizela RJ, et al. A review of the Pteropus rufus (É. Geoffroy, 1803) colonies within the Tolagnaro region of southeast Madagascar–an assessment of neoteric threats and conservation condition. Madagascar Conservation & Development. 2016;11(1):1–10. doi: 10.4314/mcd.v11i1.7

[28] Larsen PA, Hayes CE, Wilkins MA, Gomard Y, Sookhareea R, Yoder AD, et al. Population Genetics of the Mauritian Flying Fox, *Pteropus niger*. Acta Chiropterologica. 2014;16(2):293–300. doi: 10.3161/150811014X687251

[29] Woinarski JCZ, Flakus S, James DJ, Tiernan B, Dale GJ, Detto T. An Island-Wide Monitoring Program Demonstrates Decline in Reporting Rate for the Christmas Island Flying-Fox *Pteropus melanotus natalis*. Acta Chiropterologica. 2014;16(1):117–27. doi: 10.3161/150811014X683336

[30] Westcott DA, Caley P, Heersink DK, McKeown A. A state-space modelling approach to wildlife monitoring with application to flying-fox abundance. Scientific Reports. 2018;8(1):4038. doi: 10.1038/s41598-018-22294-w 29511249 PMC5840426

[31] Westcott DA, Fletcher CS, McKeown A, Murphy HT. Assessment of monitoring power for highly mobile vertebrates. Ecological Applications. 2012;22(1):374–83. doi: 10.1890/11-0132.1 22471097

[32] Westcott DA, Heersink DK, McKeown A, Caley P. Status and trends of Australia’s EPBC-listed flying-foxes. http://www.environment.gov.au/system/files/resources/9799051f-e0a2-4617-ba0d-8e2be2a364df/files/status-trends-australias-epbc-listed-flying-foxes.pdf. CSIRO, 2015.

[33] Meade J, Martin JM, Welbergen JA. Fast food in the city? Nomadic flying-foxes commute less and hang around for longer in urban areas. Behavioral Ecology. 2021;32:1151–62. doi: 10.1093/beheco/arab078

[34] Commonwealth of Australia. National Flying-fox monitoring viewer; available at https://www.environment.gov.au/webgis-framework/apps/ffc-wide/ffc-wide.jsf 2023. Available from: https://www.environment.gov.au/webgis-framework/apps/ffc-wide/ffc-wide.jsf.

[35] Ratcliffe FN. The flying-fox (Pteropus) in Australia. Melbourne 1931. 53 p.

[36] Roberts BJ, Catterall CP, Eby P, Kanowski J. Latitudinal range shifts in Australian flying-foxes: A re-evaluation. Austral ECology. 2012;37:12–22. doi: 10.1111/j.1442-9993.2011.02243.x

[37] Williams NSG, McDonnell MJ, Phelan GK, Keim LD, Van Der Ree R. Range expansion due to urbanization: Increased food resources attract Grey-headed Flying-foxes (Pteropus poliocephalus) to Melbourne. Austral Ecology. 2006;31:190–8. doi: 10.1111/j.1442-9993.2006.01590.x

[38] Boardman WSJ, David Roshier D, Reardon T, Burbidge K, McKeown A, Westcott DA, et al. Spring foraging movements of an urban population of grey-headed flying foxes (Pteropus poliocephalus). Journal of Urban Ecology. 2021;6(1):1–10. doi: 10.1093/jue/juaa034

[39] Yabsley SH, Meade J, Martin JM, Welbergen JA. Human-modified landscapes provide key foraging areas for a threatened flying mammal: The grey-headed flying-fox. PLoS ONE. 2021;16(11):1–19. doi: 10.1371/journal.pone.0259395 34723974 PMC8559981

[40] Churchill S. Australian Bats. 2 ed. Crows Nest, Australia: Allen & Unwin; 2008. 255 p.

[41] TSSC. Australian Government, Pteropus poliocephalus (Grey-headed Flying-fox). Advice to the Minister for the Environment and Heritage from the Threatened Species Scientific Committee (TSSC) on Amendments to the list of Threatened Species under the Environment Protection and Biodiversity Conservation Act 1999 (EPBC Act). http://www.environment.gov.au/biodiversity/threatened/conservation-advices/pteropus-poliocephalus. 2001.

[42] Lunney D, Richards G, Dickman C. Pteropus poliocephalus The IUCN Red List of Threatened Species 2008: e.T18751A8554062. 2008. Available from: https://www.iucnredlist.org/species/18751/8554062#conservation-actions.

[43] Tidemann CR. Grey-headed Flying-fox Pteropus poliocephalus. In: Strahan R, editor. The Mammals of Australia. Sydney, Australia: Reed New Holland; 2002. p. 439–41.

[44] Tidemann C, Nelson J. Long-distance movements of the grey-headed flying fox (Pteropus poliocephalus). Journal of Zoology. 2004;263(2):141–6. doi: 10.1017/S0952836904004960

[45] Roberts BJ, Catterall CP, Eby P, Kanowski J. Long-Distance and Frequent Movements of the Flying-Fox Pteropus poliocephalus: Implications for Management. PLOS ONE. 2012;7(8):e42532. doi: 10.1371/journal.pone.0042532 22880021 PMC3411823

[46] Yabsley SH, Meade J, Hibburt TD, Martin JM, Boardman WSJ, Nicolle D, et al. Variety is the spice of life: Flying-foxes exploit a variety of native and exotic food plants in an urban landscape mosaic. Frontiers in Ecology and Evolution. 2022;10:1–17. doi: 10.3389/fevo.2022.907966

[47] Griffith P, Parry-Jones K, Cunningham AA. Dietary Partitioning in Newly Sympatric Urban Flying-foxes (Pteropus poliocephalus and Pteropus alecto). Australian Mammalogy. 2020:A–F. doi: 10.1071/AM19047

[48] Ratcliffe F. Notes on the Fruit Bats (Pteropus spp.) of Australia. Journal of Animal Ecology. 1932;1(1):32–57.

[49] Parry-Jones K, Augee ML. Food Selection by Grey-headed Flying Foxes (Pteropus poliocephalus) Occupying a Summer Colony Site near Gosford, New South Wales. Wildlife Research. 1991;18(11):1–24.

[50] O’Brien GM. Seasonal Reproduction in Flying Foxes, Reviewed in the Context of other Tropical Mammals. Reproduction, Fertility and Development. 1993;5:499–521. doi: 10.1071/rd9930499 8190905

[51] Fox S, Spencer H, O’Brien GM. Analysis of twinning in flying-foxes (Megachiroptera) reveals superfoetation and multiple-paternity. Acta Chiropterologica. 2008;10(3):271–8. doi: 10.3161/150811008X414845

[52] Eby P. Seasonal movements of grey-headed flying-foxes Pteropus poliocephalus (Chiroptera: Pteropodidae), from two maternity camps in northern New South Wales. Wildlife Research. 1991;18:547–59.

[53] Nelson JE. Movements of Australian flying foxes (Pteropodidae: Megachiroptera). Australian Journal of Zoology. 1965;13:53–73.

[54] DECCW NSW. Draft National Recovery Plan for the Grey-headed Flying-fox Pteropus poliocephalus. Prepared by Dr Peggy Eby. Department of Environment, Climate Change and Water NSW, Sydney. 2009.

[55] Westcott DA, McKeown A, Murphy HT, Fletcher CS. A monitoring method for the grey headed flying-fox, Pteropus poliocephalus. A report to the Commonwealth Department of Sustainability, Environment, Water, Population and Communities. Tech Rep, CSIRO Ecosystem Sciences 2011.

[56] Parry-Jones KA. Historical declines since the early 1900s, and current mortality factors and abundance of the Grey-headed Flying-fox in NSW. In: Richards G, editor. Proceedings of a Workshop to Assess the Status of the Grey-headed Flying-fox in New South Wales Canberra: Australasian Bat Society; 2000. p. 57–66.

[57] Birt P. National Population Assessment Grey-headed flying foxes Pteropus poliocephalus 21 and 22 May 2005. A report to: Department of Environment and Heritage, Queensland Parks and Wildlife Service, New South Wales Department Environment and Conservation, Victorian Department Sustainability and Environment. 2005.

[58] Eby P. National Count of Grey-headed Flying foxes July 27 & 28, 2002. A report to: Environment Australia Queensland Parks and Wildlife Service NSW National Parks and Wildlife Service Victoria Dept Natural Resources and Environment. 2002.

[59] Eby P. National Count of Grey-headed Flying foxes April 12 & 13, 2003. A report to: A report to: Dept. Environment and Heritage Queensland Parks and Wildlife Service NSW Dept. Environment and Conservation Victoria Dept. Sustainability and Environment. 2003.

[60] Eby P. National Count of Grey-headed Flying-foxes April 3 & 4, 2004. A report to: Dept. Environment and Heritage Queensland Parks and Wildlife Service NSW Dept. Environment and Conservation, Victoria Dept. Sustainability and Environment. 2004:22.

[61] DAWE. National Recovery Plan for the Grey-headed Flying-fox ‘Pteropus poliocephalus’, Department of Agriculture, Water and the Environment, Canberra, March. CC BY 4.0. 2021.

[62] Australian Government. Monitoring Flying-Fox Populations: Department of Agriculture, Water and the Environment; 2020. Available from: http://www.environment.gov.au/biodiversity/threatened/species/flying-fox-monitoring.

[63] Commonwealth of Australia. Referral guideline for management actions in grey-headed and spectacled flying-fox camps. EPBC Act Policy Statement. 2015. Available from: https://www.dcceew.gov.au/environment/biodiversity/threatened/publications/referral-guideline-management-actions-flying-fox-camps.

[64] Westcott DA, McKeown A. Observer error in exit counts of flying-foxes (Pteropus spp.). Wildlife Research. 2004;31(5):551–8. doi: 10.1071/wr03091 ISI:000225660200010.

[65] Thomas L, Buckland ST, Rexstad EA, Laake JL, Strindberg S, Hedley SL, et al. Distance software: design and analysis of distance sampling surveys for estimating population size. Journal of Applied Ecology. 2010;47:5–14. doi: 10.1111/j.1365-2664.2009.01737.x 20383262 PMC2847204

[66] Jurdak R, Sommer P, Kusy B, Kottege N, Crossman C, McKeown A, et al. Camazotz: multimodal activity-based GPS sampling. In Proceedings of the 12th International Conference on Information Processing in Sensor Networks. 2013:67–78. doi: 10.1145/2461381.2461393

[67] Humbert J-Y, Mills LS, Horne JS, Dennis B. A better way to estimate population trends. Oikos. 2009;118:1940–6. doi: 10.1111/j.1600-0706.2009.17839.x

[68] Hobbs NT, Hooten MB. Bayesian Models: a Statistical Primer for Ecologists. Princeton, New Jersey: Princeton University Press; 2015.

[69] Plummer M, Stukalov A, Denwood M. Package ’rjags’: Bayesian Graphical Models using MCMC. 4–6 ed. https://cran.r-project.org/: CRAN; 2016. p. Interface to the JAGS MCMC library.

[70] R Development Core Team. R: A Language and Environment for Statistical Computing. Vienna, Austria: R Foundation for Statistical Computing; 2017.

[71] Gelman A, Rubin DB. Inference from iterative simulation using multiple sequences. Statistical Science 1992;7(4):457–72. doi: 10.1214/ss/1177011136

[72] Eby P, Roberts B, Pennay M, Welbergen JA. Pteropus poliocephalus. The IUCN Red List of Threatened Species 2021: e.T18751A22085511. 10.2305/IUCN.UK.2021-3.RLTS.T18751A22085511.en. Accessed on 25 September 2023. 2021.

[73] Eberhardt LL, Simmons MA. Assessing Rates of Increase from Trend Data. The Journal of Wildlife Management. 1992;56(3):603–10. doi: 10.2307/3808878

[74] McCarthy ED, Martin JM, Boer MM, Welbergen JA. Drone-based thermal remote sensing provides an effective new tool for monitoring the abundance of roosting fruit bats. Remote Sensing in Ecology and Conservation. 2021:1–14. doi: 10.1002/rse2.202

[75] NSW DPC. Final Report of the NSW Bushfire Inquiry 31 July 2020. Owens D, O’Kane M, editors. 2020. Available from: https://www.nsw.gov.au/departments-and-agencies/premiers-department/access-to-information/nsw-bushfire-inquiry/nsw-bushfire-inquiry-report.

[76] Reside AE, Beher J, Cosgrove AJ, Evans MC, Seabrook L, Silcock JL, et al. Ecological consequences of land clearing and policy reform in Queensland. Pacific Conservation Biology. 2017;23(3):219–30. 10.1071/PC17001.

[77] NSW Government. Native vegetation reports and resources. NSW Woody Vegetation Change 2017–18 spreadsheet Department of Planning, Industry and Environment; 2019 [28/01/2020]. Available from: https://www.environment.nsw.gov.au/topics/animals-and-plants/native-vegetation/reports-and-resources/reports.

[78] Queensland Department of Science ITaI. Land cover change in Queensland 2012–13 and 2013–14: a Statewide Landcover and Trees Study (SLATS) report. DSITI, Brisbane; 2015.

[79] Collins L, Bradstock RA, Clarke H, Clarke M, Nolan RH, Penman TD. The 2019/2020 mega-fires exposed Australian ecosystems to an unprecedented extent of high-severity fire. Environmental Research Letters. 2021;16(4):1–14. doi: 10.1088/1748-9326/abeb9e

[80] Gallagher RV, Allen S, MacKenzie BDE, Yates CD, Gosper C.R, Keith DA, et al. High fire frequency and the impact of the 2019–2020 megafires on Australian plant diversity. Diversity & Distributions. 2021;27:1166–79. doi: 10.1111/ddi.13265

[81] Baranowski K, Faust CL, Eby P, Bharti N. Quantifying the impacts of Australian bushfires on native forests and gray-headed flying foxes. Global Ecology and Conservation. 2021;27(e01566). doi: 10.1016/j.gecco.2021.e01566

[82] Welbergen JA, Booth C, Martin M. Killer climate: tens of thousands of flying foxes dead in a day. The Conversation. 2014.

[83] Evins B. Adelaide’s extreme heat kills thousands of chickens and bats: ABC; 2019 [updated 27/01/2019]. Available from: https://www.abc.net.au/news/2019-01-27/adelaide-heatwave-kills-thousands-of-bats-and-chickens/10753248.

[84] Field E, Gibson B. More than 2,000 flying foxes die from heat stress in eastern Victoria: ABC News; 2019 [updated 29/01/2019]. Available from: https://www.abc.net.au/news/2019-01-29/flying-foxes-die-from-heat-stress-eastern-victoria/10756426.

[85] Mo M, Roache M, Davies J, Hopper J, Pitty H, Foster N, et al. Estimating flying-fox mortality associated with abandonments of pups and extreme heat events during the austral summer of 2019–2020. Pacific Conservation Biology. 2022;28(2):124–39. 10.1071/PC21003.

[86] Pook EW, Gill AM, Moore PHR. Long-term Variation of Litter Fall, Canopy Leaf Area and Flowering in a *Eucalyptus maculata* Forest on the South Coast of New South Wales. Australian Journal of Botany. 1997;45(5):737–55. 10.1071/BT95063.

[87] Law B, Mackowski C, Schoer L, Tweedie T. Flowering phenology of myrtaceous trees and their relation to climatic, environmental and disturbance variables in northern New South Wales. Austral Ecology. 2000;25:160–78.

[88] Wright BR, Franklin DC, Fensham RJ. The ecology, evolution and management of mast reproduction in Australian plants. Australian Journal of Botany. 2022;70:509–30. doi: 10.1071/BT22043

[89] Parry-Jones K. Winter Flying-fox Colonies in Southern NSW. Australian Zoologist. 1985;22(2):5–6.

[90] Parry-Jones KA. Pteropus poliocephalus (Chiroptera: Pteiropodidae:) in New South Wales. Australian Mammalogy. 1987;10(2):81–5.

[91] Tait J, Perotto-Baldivieso HL, McKeown A, Westcott DA. Are Flying-Foxes Coming to Town? Urbanisation of the Spectacled Flying-Fox (Pteropus conspicillatus) in Australia. PLoS ONE. 2014;9(e109810). doi: 10.1371/journal.pone.0109810 25295724 PMC4190360

[92] Mo M, Mark M, Edward E, Vanessa P, Welbergen JA. A report of direct mortality in grey-headed flying-foxes (Pteropus poliocephalus) from the 2019–2020 Australian megafires. Australian Mammalogy 2022;44(3):419–22. doi: 10.1071/AM21041

[93] Myerscough PJ. Ecology of Myrtaceae with special reference to the Sydney region. Cunninghamia. 1998;5(4):787–807.

[94] Collins L. Eucalypt forests dominated by epicormic resprouters are resilient to repeated canopy fires. Journal of Ecology. 2020;108:310–24. doi: 10.1111/1365-2745.13227

[95] Tidemann CR, Nelson J. Life expectancy, causes of death and movements of the grey-headed flying-fox (Pteropus poliocephalus) inferred from banding. Acta Chiropterologica. 2011;13(2):419–29. doi: 10.3161/150811011X624901

[96] Dury GH. High Temperature Extremes in Australia. Annals of the Association of American Geographers. 1972;62(3):388–400. doi: 10.1111/j.1467-8306.1972.tb00871.x

[97] News ABC. Heat records around Australia continue to tumble, with Canberra reaching 44 degrees and Penrith 48.9 2020 [updated 05/01/202021/01/2020]. Available from: https://www.abc.net.au/news/2020-01-04/heat-record-canberra-penrith-fire-bushfire-bureau-of-meteorology/11841014.

[98] Scopelianos S, Slessor C. Adelaide now hottest capital city on record as temperatures soar throughout SA 2019 [updated 24/01/201921/01/2020]. Available from: https://www.abc.net.au/news/2019-01-24/sa-heating-up-with-records-expected-to-be-broken/10745220.

[99] Bureau of Meteorology. Special Climate Statement 68—widespread heatwaves during December 2018 and January 2019 Commonwealth of Australia: 2019.

[100] Australian Government Bureau of Meteorology. Special Climate Statement 72—dangerous bushfire weather in spring 2019. 2019 18/12/2019. Report No.

[101] Australian Government Bureau of Meteorology. Special Climate Statement 71—severe fire weather conditions in southeast Queensland and northeast New South Wales in September 2019. 2019 24/09/2019. Report No.

[102] Bradstock RA, Williams JE, Gill AM. Flammable Australia: the fire regimes and biodiversity of a continent. Bradstock RA, Williams JE, Gill AM, editors. United Kingdom: Cambridge University Press; 2002. 462 p.

[103] Bradstock RA, Gill AM, Williams JE. Flammable Australia: fire regimes, biodiversity and ecosystems in a changing world. Bradstock RA, Gill AM, Williams JE, editors. Collingwood, Australia: CSIRO Publishing; 2012.

[104] Williams RJ, Bradstock RA, Cary GJ, Enright NJ, Gill AM, Liedloff AC, et al. Interactions between climate change, fire regimes and biodiversity in Australia—a preliminary report. Report to the Department of Climate Change and Department of the Environment, Water, Heritage and the Arts, Canberra. 2009.

[105] Gill AM, Woinarski JCZ, York A. Australia’s Biodiversity–Responses to fire: Plants, birds and invertebrates—Biodiversity Technical Paper, No. 1. Canberra: Department of the Environment and Heritage; 1999. 266 p.

[106] Eby P, Lunney D. Managing the Grey-headed Flying-fox as a Threatened Species in NSW. Bradstock RA, Williams JE, Gill AM, editors. Mosman NSW: Royal Zoological Society of New South Wales; 2002. 286 p.

[107] Whelan RJ, Rodgerson L, Dickman CR, Sutherland E. Critical life cycles of plants and animals: developing a process-based understanding of population changes in fire-prone landscapes. In: Bradstock RA, Williams JE, Gill AM, editors. Flammable Australia: the fire regimes and biodiversity of a continent. United Kingdom: Cambridge University Press; 2002. p. 94–124.

[108] Gill AM. Bushfires and biodiversity in southern Australian forests. In: Bradstock RA, Gill AM, Williams JE, editors. Flammable Australia: fire regimes, biodiversity and ecosystems in a changing world. Collingwood, Australia: CSIRO Publishing; 2012. p. 235–52.

[109] Voigt CC, Kingston T. Bats in the Anthropocene. In: Voigt CC, Kingston T, editors. Bats in the Anthropocene: Conservation of Bats in a Changing World. Switzerland: Springer Open; 2016. p. 1–9.

[110] Garnett ST, Lindenmayer DB. Conservation science must engender hope to succeed. Trends in Ecology and Evolution. 2011;26(2):59–60. doi: 10.1016/j.tree.2010.11.009 21185618

[111] Swaisgood RR, Sheppard JK. The Culture of Conservation Biologists: Show Me the Hope! BioScience. 2010;60(8):626–30. doi: 10.1525/bio.2010.60.8.8

[112] Jones M, Abatzoglou J, Veraverbeke S, Andela N, Lasslop G, Forkel M, et al. Global and Regional Trends and Drivers of Fire Under Climate Change. Reviews of Geophysics. 2022;60. doi: 10.1029/2020RG000726

[113] Sharples JJ, Cary GJ, Fox-Hughes P, Mooney S, Evans JP, Fletcher M-S, et al. Natural hazards in Australia: extreme bushfire. Climatic Change. 2016;139(1):85–99. doi: 10.1007/s10584-016-1811-1

[114] Canadell JG, Meyer CP, Cook GD, Dowdy A, Briggs PR, Knauer J, et al. Multi-decadal increase of forest burned area in Australia is linked to climate change. Nature Communications. 2021;12(1):6921. doi: 10.1038/s41467-021-27225-4 34836974 PMC8626427

[115] Clarke HG, Smith PL, Pitman AJ. Regional signatures of future fire weather over eastern Australia from global climate models %J International Journal of Wildland Fire. 2011;20(4):550–62. 10.1071/WF10070.

[116] Meade J, Van Der Ree R, Stepanian PM, Westcott DA, Welbergen JA. Using weather radar to monitor the number, timing and directions of flying-foxes emerging from their roosts. Scientific Reports. 2019;9:1–10. doi: 10.1038/s41598-019-46549-2 31308411 PMC6629676

[117] Kreutzfeldt J, Floeter C, Lingner T, Schmitz-Beuting L, Reich M, Kunz VD. Analytical volume model for optimized spatial radar bat detection in onshore wind parks. PLoS ONE. 2020;15(e0239911):1–22. doi: 10.1371/journal.pone.0239911 32997717 PMC7526923

[118] Bruderer B, Steuri T, Aschwanden J, Liechti F. Vom militärischen Zielfolgeradar zum Vogelradar. Der Ornithologische Beobachter. 2012;109(3):157–76.

