# Supplementary figures and images for: Population trends in the vulnerable Grey-headed flying-fox, Pteropus poliocephalus; results from a long-term, range-wide study
Source: PLoS One. 2024 Mar 21;19(3):e0298530. doi: 10.1371/journal.pone.0298530 (PMC10956843; doi:10.1371/journal.pone.0298530)

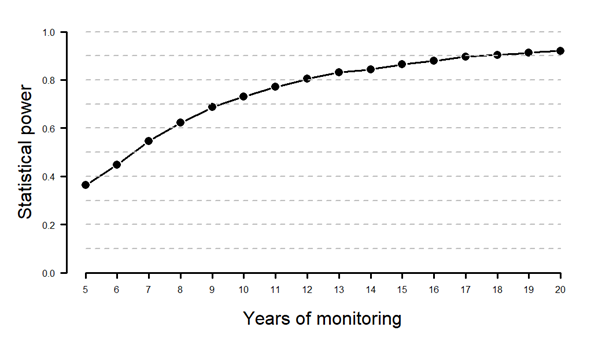

Supplement: S1 Fig — Results are derived from log-linear modelling of simulated total national counts of grey-headed flying foxes, where simulations assume an underlying decrease of 0.036% reduction / year, though the realised population growth is subject to process noise (see text for details). (TIF) [file pone.0298530.s001.tif]

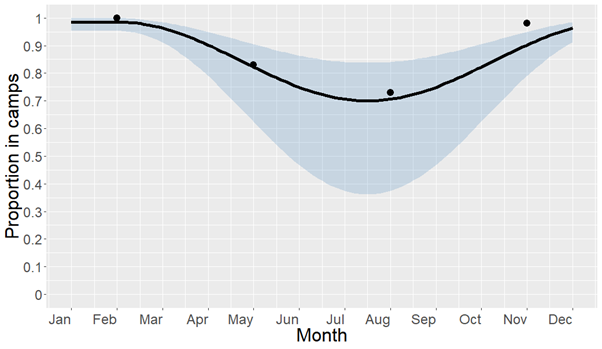

Supplement: S2 Fig — The solid line represents the mean, and shading the 95% credibility interval. Observed datapoints represent the proportions of radio-tracked individuals within camp [32]. (TIF) [file pone.0298530.s002.tif]

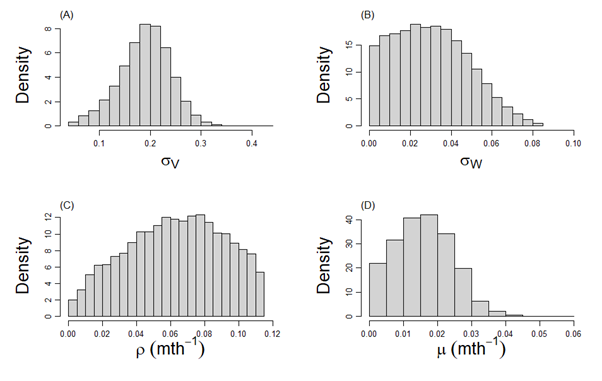

Supplement: S3 Fig — Posterior distributions for (A). Standard deviation of estimated log-normal count error, (B). Standard deviation of monthly process noise in population change, (C). Rate of recruitment into population during recruitment months (December, January, February), and (D) monthly mortality rate (year-round). (TIF) [file pone.0298530.s003.tif]

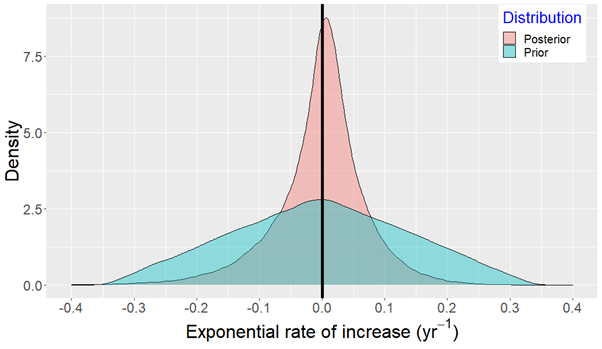

Supplement: S4 Fig — (TIF) [file pone.0298530.s004.tif]
